# Advancements and challenges in triple-negative breast cancer: a comprehensive review of therapeutic and diagnostic strategies

**DOI:** 10.3389/fonc.2024.1405491

**Published:** 2024-05-28

**Authors:** Nating Xiong, Heming Wu, Zhikang Yu

**Affiliations:** ^1^ Department of Blood Transfusion, Meizhou People’s Hospital, Meizhou Academy of Medical Sciences, Meizhou, China; ^2^ Meizhou Municipal Engineering and Technology Research Centre for Molecular Diagnostics of Major Genetic Disorders, Meizhou People’s Hospital, Meizhou, China; ^3^ Research Experiment Centre, Meizhou People’s Hospital, Meizhou Academy of Medical Sciences, Meizhou, China; ^4^ Guangdong Engineering Technological Research Centre of Clinical Molecular Diagnosis and Antibody Drugs, Meizhou People’s Hospital, Meizhou Academy of Medical Sciences, Meizhou, China

**Keywords:** triple-negative breast cancer, therapeutic challenges, diagnostic challenges, targeted therapies, immunotherapy, precision medicine

## Abstract

Triple-negative breast cancer (TNBC) poses significant challenges in oncology due to its aggressive nature, limited treatment options, and poorer prognosis compared to other breast cancer subtypes. This comprehensive review examines the therapeutic and diagnostic landscape of TNBC, highlighting current strategies, emerging therapies, and future directions. Targeted therapies, including PARP inhibitors, immune checkpoint inhibitors, and EGFR inhibitors, hold promise for personalized treatment approaches. Challenges in identifying novel targets, exploring combination therapies, and developing predictive biomarkers must be addressed to optimize targeted therapy in TNBC. Immunotherapy represents a transformative approach in TNBC treatment, yet challenges in biomarker identification, combination strategies, and overcoming resistance persist. Precision medicine approaches offer opportunities for tailored treatment based on tumor biology, but integration of multi-omics data and clinical implementation present challenges requiring innovative solutions. Despite these challenges, ongoing research efforts and collaborative initiatives offer hope for improving outcomes and advancing treatment strategies in TNBC. By addressing the complexities of TNBC biology and developing effective therapeutic approaches, personalized treatments can be realized, ultimately enhancing the lives of TNBC patients. Continued research, clinical trials, and interdisciplinary collaborations are essential for realizing this vision and making meaningful progress in TNBC management.

## Introduction

Triple-negative breast cancer (TNBC) represents a formidable challenge in the field of oncology, characterized by its aggressive behavior, limited treatment options, and poorer prognosis compared to other breast cancer subtypes (1). TNBC is defined by the absence of estrogen receptor (ER), progesterone receptor (PR), and human epidermal growth factor receptor 2 (HER2) expression, making it unresponsive to targeted therapies commonly employed in other breast cancer subtypes (2). Despite advances in breast cancer research and treatment, TNBC remains a significant cause of morbidity and mortality worldwide (3). TNBC accounts for approximately 15–20% of all diagnosed breast cancers, representing a heterogeneous group of Tumors with distinct molecular characteristics and clinical behaviors (4). It is more prevalent among younger women, African American women, and those with BRCA1 mutations, highlighting the importance of genetic predisposition in TNBC development (5, 6). Furthermore, TNBC tends to present at a more advanced stage, with a higher likelihood of early metastasis to distant organs such as the lungs, liver, and brain, contributing to its poorer prognosis compared to other breast cancer subtypes (7). TNBC exhibits molecular heterogeneity, comprising several distinct subtypes with varying biological features and treatment responses (8). Basal-like TNBC, characterized by the expression of basal cytokeratins (CK5/6, CK14, CK17), is the most common subtype and shares molecular similarities with basal-like breast cancers (9). Other subtypes include mesenchymal, immunomodulatory, and luminal androgen receptor (LAR) subtypes, each with unique molecular signatures and clinical implications (10). The pathogenesis of TNBC involves the dysregulation of multiple signaling pathways, including the PI3K/AKT/mTOR, MAPK/ERK, and JAK/STAT pathways, driving Tumor proliferation, invasion, and metastasis (11). Accurate diagnosis of TNBC is crucial for guiding treatment decisions (12) however, challenges exist in distinguishing TNBC from other breast cancer subtypes and assessing Tumor heterogeneity within TNBC Tumors (13). Immunohistochemistry (IHC) is commonly used to determine ER, PR, and HER2 status; however, discordance between IHC and gene expression profiling highlights the limitations of current diagnostic methods. Moreover, intratumoral heterogeneity and the presence of rare subpopulations within TNBC Tumors pose additional diagnostic challenges, necessitating the development of more precise and comprehensive diagnostic tools (14). TNBC management relies primarily on a multimodal approach, including chemotherapy, surgery, and radiation therapy, due to the lack of specific targeted therapies. Neoadjuvant chemotherapy is often administered to downstage Tumors and increases the likelihood of breast-conserving surgery, followed by adjuvant chemotherapy to reduce the risk of recurrence (15). Despite initial response rates, a significant proportion of TNBC patients experience disease recurrence and metastasis, highlighting the need for novel therapeutic strategies. The landscape of TNBC treatment is rapidly evolving, driven by advances in molecular biology, immunotherapy, and precision medicine. Emerging targeted therapies, including PARP inhibitors, immune checkpoint inhibitors, and tyrosine kinase inhibitors, hold promise for improving outcomes in TNBC patients (16). Furthermore, the integration of genomic profiling, liquid biopsy, and novel imaging techniques may enable personalized treatment approaches tailored to individual Tumor biology and patient characteristics (17). Collaborative efforts among researchers, clinicians, and pharmaceutical companies are essential to overcome the therapeutic and diagnostic challenges associated with TNBC and improve patient outcomes. TNBC represents a complex and heterogeneous disease entity associated with unique therapeutic and diagnostic challenges. Despite significant progress in breast cancer research and treatment, TNBC remains a formidable clinical problem, underscoring the urgent need for innovative therapeutic approaches and precision medicine strategies. By addressing these challenges collaboratively, the clinical community can strive towards improved outcomes and better quality of life for patients with TNBC.

## Molecular subtypes and pathogenesis of TNBC

TNBC is characterized by its molecular heterogeneity, encompassing several distinct subtypes with varying biological features and clinical behaviors. Understanding these molecular subtypes and the underlying pathogenesis is crucial for guiding treatment decisions and improving patient outcomes. In 2011, Lehmann et al. conducted gene expression analysis of TNBC cases, leading to the description of seven possible subtypes within this breast cancer type (18). Later in 2016, Lehmann et al. revised this classification based on histologic evaluation, laser microdissection, and gene expression analysis of TNBCs (19). The revised classification identified four tumor-specific subtypes: basal-like (BL; BL1 and BL2), mesenchymal, Immunomodulatory subtype and luminal androgen receptor (LAR) subtypes (**Table 1**). BL 5subtypes predominate in TNBC (up to 80% of cases), while mesenchymal and LAR subtypes occur less frequently (20).

**Table 1:** Molecular subtypes of triple-negative breast cancer.

Subtype	Molecular Features	Clinical Characteristics
Basal-like	Expression of basal cytokeratins (CK5/6, CK14, CK17), TP53 mutations	High histological grade, aggressive phenotype, poor prognosis
Mesenchymal	Upregulation of genes associated with epithelial-to-mesenchymal transition (EMT)	Increased motility, invasiveness, resistance to therapy
Immunomodulatory	Upregulation of immune response genes, enrichment of tumor-infiltrating lymphocytes (TILs)	Better prognosis, higher response rates to immunotherapy
Luminal Androgen Receptor (LAR)	Expression of androgen receptor (AR), luminal-associated genes	Less proliferative, luminal-like features, may have better prognosis

Basal-like Subtype: The basal-like subtype is the most common molecular subtype of TNBC, accounting for approximately 70–80% of cases (21). It is characterized by the expression of basal cytokeratins (CK5/6, CK14, CK17) and shares molecular similarities with basal-like breast cancers. Basal-like TNBC Tumors typically exhibit high histological grade, increased proliferation, and frequent TP53 mutations (22). The molecular similarities between basal-like triple-negative breast cancer (TNBC) and basal-like breast cancers extend beyond their shared expression of basal cytokeratins. Both subtypes exhibit a characteristic gene expression profile typified by high levels of genes associated with basal epithelial cells, such as cytokeratins 5, 14, and 17. Additionally, they often demonstrate downregulation of genes related to luminal epithelial differentiation, such as estrogen receptor (ER), progesterone receptor (PR), and human epidermal growth factor receptor 2 (HER2).Clinically, patients with basal-like TNBC tend to have a poorer prognosis and higher rates of metastasis compared to other TNBC subtypes. It is typically identified through the expression of specific immunohistochemical markers, including cytokeratins (CK5, CK6, CK14, or CK17), EGFR, SMA, P-cadherin, p63, or c-kit antigen. Additionally, this molecular subtype is characterized by the absence of ERα, PgR, HER2, or “luminal” cytokeratins (CK8/18/19). Moreover, basal-like breast cancer exhibits a higher mitotic index along with increased expression of Ki-67 and p53 (23). This subtype commonly demonstrates heightened genome instability and inactivation of the Rb pathway. Furthermore, it often displays upregulation of genes associated with proliferation, such as cyclin E1, BUB1, topoisomerase IIα, CDC2, and PCNA (24).

Mesenchymal Subtype: The mesenchymal subtype of TNBC is characterized by the expression of genes associated with epithelial-to-mesenchymal transition (EMT), such as vimentin, fibronectin, and Snail (25). EMT comprises three types with distinct roles: type I aids embryonic morphogenesis; type II responds to inflammation, as in wound healing and tissue regeneration; type III drives metastasis, the leading cause of cancer mortality (26). EMT serves as a key feature of cancer invasion, involving a transition from epithelial to mesenchymal cell phenotypes. This transition is regulated by multiple signaling pathways, including TGF-β, Notch, and Wnt, and influenced by factors like hypoxia and microRNAs. Converging on transcription factors such as Snail, Slug, and Twist, these pathways collectively promote the EMT process [24]. These Tumors display mesenchymal features, including increased motility, invasiveness, and resistance to therapy. Mesenchymal TNBC Tumors often exhibit molecular signatures resembling mesenchymal stem cells and are associated with a more aggressive phenotype and worse clinical outcomes.

Immunomodulatory Subtype: The immunomodulatory subtype of TNBC is characterized by the upregulation of genes involved in immune response pathways, including lymphocyte activation, antigen presentation, and cytokine signaling (27). These Tumors are enriched with Tumor-infiltrating lymphocytes (TILs) and exhibit an immune-active microenvironment (28). Immunomodulatory TNBC Tumors often have a better prognosis and higher response rates to immunotherapy compared to other TNBC subtypes (29). Burstein et al. classified another basal-like subtype, BLIA, characterized by the upregulation of immune regulation pathways. In contrast to BLIS, BLIA Tumors show elevated expression of genes involved in B cell, T cell, and natural killer cell functions. BLIA subtype demonstrates a favorable prognosis, with activation of STAT transcription factor-mediated pathways and high expression of STAT genes (30).

Luminal Androgen Receptor (LAR) Subtype: The LAR subtype of TNBC is characterized by the expression of androgen receptor (AR) and luminal-associated genes, such as FOXA1 and GATA3 (31). These Tumors often display luminal-like features and are less proliferative compared to other TNBC subtypes (32). LAR TNBC Tumors are more commonly found in older women and may exhibit a less aggressive clinical course compared to basal-like TNBC Tumors. LAR exhibit high expression of luminal cytokeratins (CK7/8, CK18, and CK19) and lack basal cytokeratin’s (CK5/6, CK14, and CK17), distinguishing them from other subtypes. The luminal androgen receptor (LAR) subtype, characterized by CK7/8, CK18, and CK19 positivity, demonstrates a more favorable prognosis compared to basal phenotype markers. Furthermore, the LAR subtype is characterized by mutations in the PI3K pathway, as reported by Lehmann et al., who identified mutations in genes such as PIK3CA, KMT2C, and CDH1 (33). Bareche et al. also observed a higher mutation load, particularly in PI3KCA, AKT, and CDH1 genes. The presence of PIK3CA mutations is particularly notable in LAR subtype TNBC (34). Studies on TNBC cell lines, such as MDA-MB-231 and MDA-MB-453, have highlighted the role of exosomes enriched with CD151 protein in cancer progression. Li SP et al. demonstrated that CD151-enriched exosomes in MDA-MB-231 cells contribute to cell migration and tumor invasion, suggesting their importance as mediators of cancer progression (35). Similarly, Li D et al. investigated the molecular mechanisms underlying cancer progression in the LAR subtype of TNBC using the MDA-MB-453 cell line. They found that CD151-enriched exosomes contribute to the invasive potential of malignant tumor cells, suggesting CD151 as a potential target for LAR subtype TNBC treatment (36).

The pathogenesis of TNBC involves the dysregulation of multiple signaling pathways, contributing to Tumor initiation, progression, and metastasis. Key pathways implicated in TNBC pathogenesis include:

PI3K/AKT/mTOR Pathway: Dysregulation of the phosphatidylinositol 3-kinase (PI3K) pathway is commonly observed in TNBC, leading to increased cell proliferation, survival, and invasion. Activation of AKT and mammalian target of rapamycin (mTOR) signaling promotes Tumor growth and metastasis in TNBC (37).

MAPK/ERK Pathway: Aberrant activation of the mitogen-activated protein kinase (MAPK) pathway, particularly the extracellular signal-regulated kinase (ERK) pathway, is implicated in TNBC pathogenesis. Dysregulated MAPK/ERK signaling promotes cell proliferation, survival, and metastasis in TNBC Tumors. JAK/STAT Pathway: Dysregulation of the Janus kinase (JAK)/signal transducer and activator of transcription (STAT) pathway contributes to TNBC tumorigenesis and progression. Activation of JAK/STAT signaling promotes cell proliferation, invasion, and immune evasion in TNBC. The molecular subtypes and pathogenesis of TNBC are diverse and complex, reflecting the heterogeneous nature of this aggressive disease. Further research into the molecular drivers of TNBC and their clinical implications is essential for developing targeted therapies and improving patient outcomes (38).

## Diagnostic challenges in TNBC

**Figure 1:**
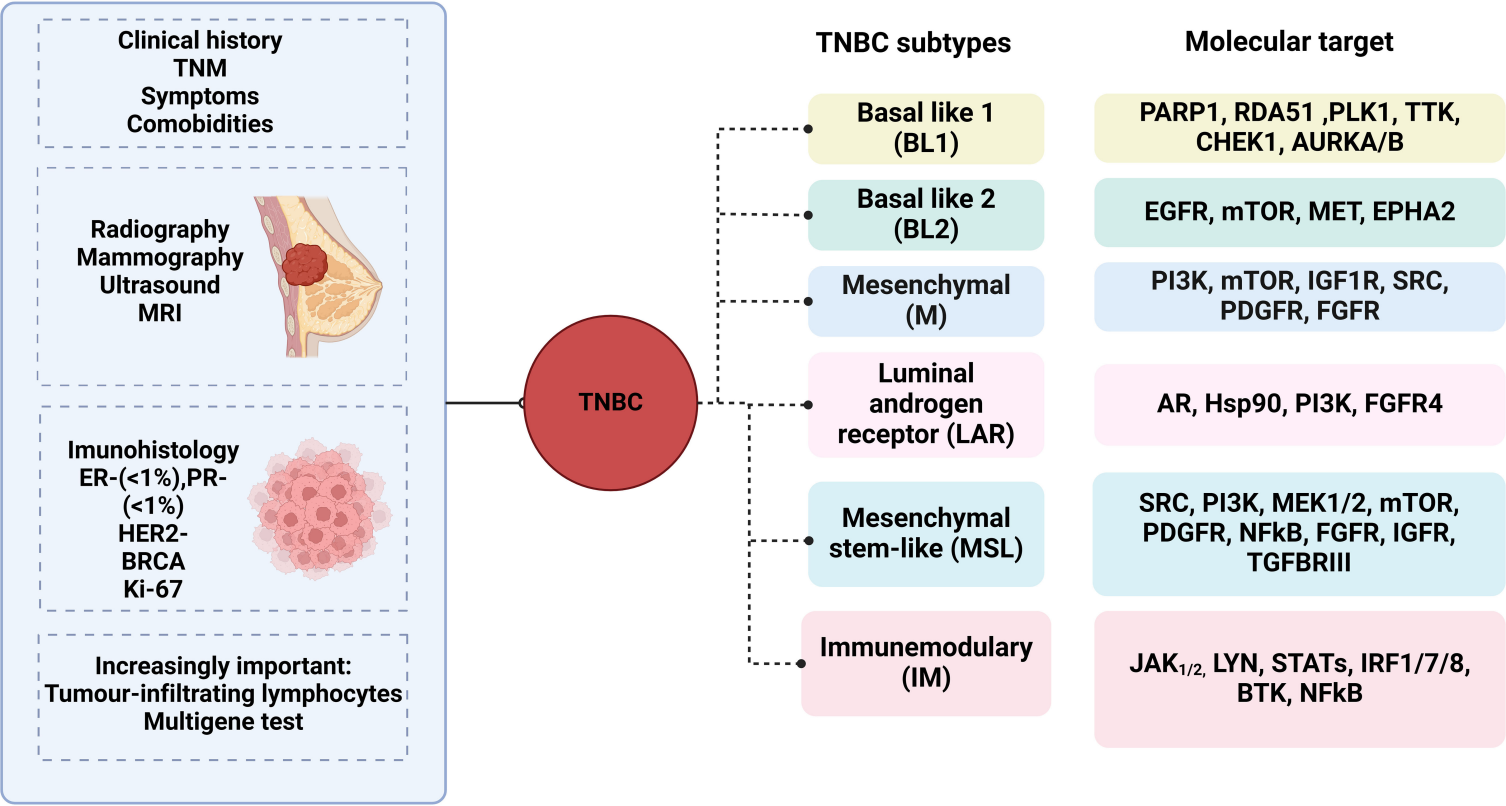
Diagnostic Challenges in Triple negative breast cancer. figure showing the diagnostic challenges in TNBC, including immunohistochemistry, gene expression profiling, and molecular imaging techniques.

Accurate diagnosis of triple-negative breast cancer (TNBC) is essential for guiding treatment decisions and predicting patient outcomes. However, several challenges exist in the diagnosis of TNBC, including the identification of specific biomarkers, assessment of Tumor heterogeneity, and differentiation from other breast cancer subtypes (**Figure 1**).

Immunohistochemistry (IHC): Immunohistochemistry is commonly used to determine the expression of estrogen receptor (ER), progesterone receptor (PR), and human epidermal growth factor receptor 2 (HER2) in breast cancer tissues (39). TNBC is defined by the absence of ER, PR, and HER2 expression; however, interpretation of IHC results can be challenging due to interobserver variability and discordance between IHC and gene expression profiling. Moreover, heterogeneous expression of hormone receptors within TNBC Tumors may lead to sampling errors and misclassification of Tumor subtypes (40).

Gene Expression Profiling: Gene expression profiling assays, such as PAM50 and the Breast Cancer Index, provide molecular subtype classification and prognostic information in breast cancer (41). These assays can distinguish between luminal, HER2-enriched, and basal-like subtypes, including TNBC. However, access to gene expression profiling assays may be limited in clinical practice, and their utility in guiding treatment decisions for TNBC remains under investigation. The PAM50 assay, initially described by Perou et al., is a gene expression-based tool utilized for molecular profiling of breast cancer. It categorizes breast cancer into intrinsic subtypes by evaluating the expression patterns of 50 genes alongside 8 housekeeping genes (42). Employing the Nano String method, the PAM50 assay accurately measures gene activity levels within breast Tumor samples. This assay furnishes critical insights into the molecular subtype of breast cancer, aiding clinicians in treatment decisions and prognostication (43). Notably, the risk of recurrence (ROR) score derived from the PAM50 assay has demonstrated prognostic utility in early-stage luminal breast cancer, supported by studies such as Trans ATAC and ABCSG-8 trials (44).

Molecular Imaging Techniques: Molecular imaging techniques, such as positron emission tomography (PET) and magnetic resonance imaging (MRI), play a role in TNBC diagnosis and staging. PET imaging with radiotracers targeting glucose metabolism (e.g., 18F-fluorodeoxyglucose) can detect primary Tumors and metastatic lesions in TNBC patients (45). Similarly, dynamic contrast-enhanced MRI provides functional information about Tumor vascularity and can aid in treatment planning. However, these imaging modalities may not reliably distinguish between TNBC and other breast cancer subtypes or assess Tumor heterogeneity (46).

Diagnostic challenges require a multidisciplinary approach and integration of complementary diagnostic modalities. Emerging technologies, such as liquid biopsy and next-generation sequencing, hold promise for improving the accuracy and precision of TNBC diagnosis. Liquid biopsy enables the detection of circulating Tumor cells (CTCs) and cell-free DNA (cfDNA) in peripheral blood, providing real-time information about Tumor dynamics and treatment response (47–49). Similarly, next-generation sequencing allows comprehensive molecular profiling of TNBC Tumors, identifying actionable genetic alterations and guiding targeted therapy selection. accurate diagnosis of TNBC remains a clinical challenge due to the complexity of Tumor biology and the limitations of current diagnostic tools (50). Addressing these challenges requires ongoing research efforts and collaboration among clinicians, pathologists, and researchers to develop more reliable and comprehensive diagnostic strategies for TNBC patients.

## Therapeutic strategies for TNBC

**Figure 2:**
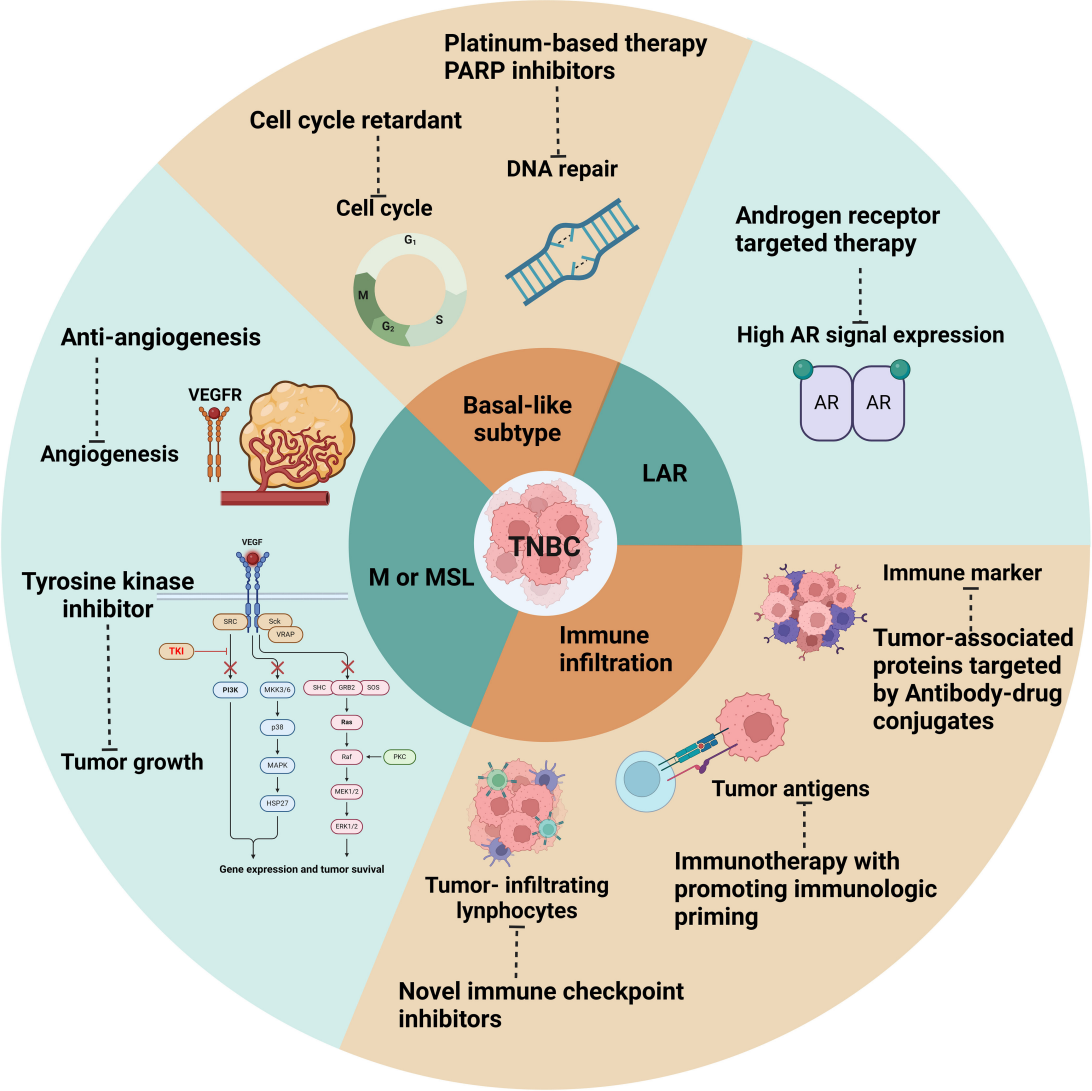
Therapeutic Strategies for Triple negative breast cancer. figure depicting the therapeutic strategies for TNBC, including chemotherapy, surgery, and radiation therapy, along with emerging targeted therapies and immunotherapeutic agents.

Triple-negative breast cancer (TNBC) poses significant therapeutic challenges due to the absence of specific molecular targets, such as estrogen receptor (ER), progesterone receptor (PR), and human epidermal growth factor receptor 2 (HER2), which are commonly targeted in other breast cancer subtypes. As a result, TNBC management relies primarily on a multimodal approach, including chemotherapy, surgery, and radiation therapy, along with emerging targeted therapies and immunotherapeutic agents (**Figure 2**).

### Chemotherapy

Chemotherapy plays a crucial role in the management of triple-negative breast cancer (TNBC), a subtype known for its aggressive behavior and limited treatment options (51). Various chemotherapeutic agents and regimens have been explored in both the neoadjuvant and adjuvant settings to improve outcomes for patients with TNBC. In the neoadjuvant setting, chemotherapy serves to shrink Tumors before surgery, allowing for more conservative surgical approaches and potentially improving long-term outcomes. Anthracyclines, such as doxorubicin and epirubicin, combined with cyclophosphamide (AC) have been widely used in TNBC due to their efficacy in inducing tumor regression. Studies have reported higher response rates to neoadjuvant AC-based chemotherapy in TNBC compared to other subtypes (52). Intensifying conventional AC chemotherapy regimens has also been shown to enhance response rates in TNBC patients (53). Taxanes, including paclitaxel and docetaxel, are another important class of chemotherapeutic agents used in TNBC. The addition of taxanes to anthracycline-based regimens in the neoadjuvant setting has been associated with increased pathologic complete response (pCR) rates, which in turn predicts improved long-term outcomes for TNBC patients (54). Platinum agents, such as cisplatin and carboplatin, have garnered significant interest in TNBC treatment, particularly in tumors with BRCA1 mutations or DNA repair deficiencies. Preoperative therapy with platinum agents has shown promising results in inducing pCR in TNBC patients, especially those with BRCA1 mutations. Studies are ongoing to further evaluate the role of platinum agents in both neoadjuvant and metastatic settings (55). In addition to traditional chemotherapeutic agents, novel agents and combination therapies are being investigated for TNBC treatment. PARP inhibitors, which exploit DNA repair defects in BRCA-mutated tumors, have shown efficacy in preclinical studies and early-phase clinical trials. Furthermore, novel microtubule-stabilizing agents like ixabepilone and nanoparticle albumin-bound paclitaxel (nab-paclitaxel) are being explored for their potential in overcoming resistance to traditional taxanes in TNBC (56). In the metastatic setting, chemotherapy remains a cornerstone of treatment for TNBC patients. Taxanes continue to be commonly used, although studies have shown mixed results regarding their specific benefit in TNBC compared to other subtypes (57). Platinum agents have emerged as a promising option for metastatic TNBC, particularly in patients with BRCA mutations or DNA repair deficiencies. Chemotherapy remains an essential component of treatment for TNBC across all stages of the disease. Ongoing research efforts aim to optimize chemotherapy regimens, identify predictive biomarkers, and explore novel therapeutic strategies to improve outcomes for patients with this aggressive subtype of breast cancer.

### Surgery

Surgery remains a cornerstone of TNBC treatment, with the primary goal of achieving complete Tumor resection and local disease control. Options include breast-conserving surgery (lumpectomy) or mastectomy, depending on Tumor size, location, and patient preference (58). Sentinel lymph node biopsy or axillary lymph node dissection may be performed to assess lymph node involvement and guide adjuvant therapy decisions. In some cases, neoadjuvant chemotherapy may be administered to downstage Tumors and facilitate breast-conserving surgery.

### Radiation therapy

It plays a crucial role in the treatment of breast cancer, including TNBC. TNBC tends to be more aggressive and lacks specific targeted therapies, making radiotherapy an essential component of multidisciplinary treatment (59). Studies have shown that preoperative radiotherapy is feasible and well-tolerated in localized breast cancer, with complete pathological response rates ranging from 10% to 26% (60). The benefits of preoperative radiotherapy include reducing the risk of recurrence and improving survival rates. Additionally, preoperative radiotherapy has been associated with low toxicity profiles and no disadvantages compared to adjuvant radiotherapy (61). In the context of TNBC, preoperative radiotherapy has been studied as part of neoadjuvant radio chemotherapy regimens. While concurrent chemoradiotherapy is more common in other cancers, the combination has shown promise in TNBC, particularly in aggressive subtypes. Studies have demonstrated varying degrees of pathological complete response (pCR) rates with preoperative chemo-radiotherapy in TNBC, ranging from 23% to 71% (62). Toxicity profiles have generally been manageable, with some studies reporting mild to moderate acute toxicity. Furthermore, the use of moderate hypofractionation schedules in preoperative radiotherapy has shown promising results, with acceptable toxicity profiles and similar rates of postoperative complications compared to conventional fractionation. The preoperative radiotherapy, either alone or in combination with chemotherapy, holds potential as a valuable treatment approach for TNBC, offering benefits in terms of local control and survival outcomes while maintaining manageable toxicity profiles. However, further research is needed to optimize treatment protocols and better understand the long-term effects of preoperative radiotherapy in TNBC.

### Emerging targeted therapies

Recent advances in understanding TNBC biology have led to the identification of potential therapeutic targets, offering new avenues for targeted therapy development. Poly (ADP-ribose) polymerase (PARP) inhibitors, such as olaparib and talazoparib, have shown promise in TNBC patients with BRCA1/2 mutations, leading to FDA approval for certain patients with germline BRCA mutations. Additionally, inhibitors targeting other DNA repair pathways, such as ATR and CHK1, are under investigation in clinical trials for TNBC (63) (**Table 2**). PARP inhibitors represent a significant advancement in the treatment of breast cancer, particularly in patients with TNBC who harbor BRCA1/2 mutations (64). By exploiting the defective DNA repair mechanisms in BRCA-mutated Tumors, PARP inhibitors disrupt DNA damage repair pathways, leading to synthetic lethality and ultimately Tumor cell death (65). Clinical trials, such as OlympiAD and EMBRACA, have demonstrated the efficacy of PARP inhibitors such as Olaparib and talazoparib in improving progression-free survival and objective response rates in advanced or metastatic TNBC patients with BRCA mutations (66). Notably, talazoparib has shown particularly promising results, with longer progression-free survival and higher objective response rates compared to chemotherapy (67). Furthermore, ongoing research, including trials like OlympiA and NEOTALA, continues to explore the potential benefits of PARP inhibitors in early-stage TNBC and combination therapies to overcome clinical resistance, promising a brighter future for patients with this aggressive subtype of breast cancer (68). Understanding the mechanisms of resistance to PARP inhibitors and developing strategies to overcome them will be crucial in maximizing the efficacy of these agents in TNBC treatment (69). Androgen Receptor inhibitors show promise in treating TNBC, particularly the LAR subtype. They have demonstrated efficacy in clinical trials, with drugs like enzalutamide and abiraterone showing favorable outcomes in terms of progression-free survival (PFS) and overall survival (OS). Combinations with other agents, such as PARP inhibitors or CDK inhibitors, have shown synergistic effects in preclinical studies (70). Ongoing research aims to validate these findings in larger clinical trials and explore combination therapies further. Cyclin-Dependent Kinase (CDK) inhibitors, like palbociclib and ribociclib, have shown promise in TNBC treatment, particularly for the LAR subtype. They inhibit Tumor cell proliferation and have demonstrated efficacy both as monotherapy and in combination with other targeted drugs in preclinical studies (71). Clinical trials have shown improvements in PFS and OS in patients with ER+/HER2− breast cancer. However, further research is needed to optimize their use in TNBC therapy. Inhibitors of the PI3K/AKT/mTOR signaling pathway offer a prospective approach for treating TNBC, given the pathway’s role in tumor cell proliferation and survival (72). Preclinical studies have shown their potential to suppress tumor growth and induce apoptosis in TNBC cells. Mutations in PIK3CA, prevalent in TNBC, make this pathway an attractive target for therapy (73). Ongoing research aims to further elucidate the efficacy of these inhibitors and explore combination therapies to improve patient outcomes. Tyrosine kinase inhibitors (TKIs) target aberrant signaling pathways in TNBC, including the epidermal growth factor receptor (EGFR) and vascular endothelial growth factor receptor (VEGFR) pathways (74). Clinical trials investigating EGFR inhibitors, such as cetuximab and erlotinib, in combination with chemotherapy have shown mixed results in TNBC patients, highlighting the need for further research to identify predictive biomarkers and optimal treatment strategies.

**Table 2:** Emerging targeted therapies, mechanisms and clinical trials for TNBC.

Target	Therapeutic Agent	Mechanism of Action	Clinical Trials and Results
Poly (ADP-ribose) polymerase (PARP)	Olaparib, Talazoparib, Niraparib	Inhibits PARP enzyme, leading to DNA damage and cell death	Phase III trials (OlympiAD, EMBRACA) demonstrated improved progression-free survival in BRCA-mutated TNBC
Androgen receptor (AR)	Enzalutamide, Bicalutamide	Blocks androgen receptor signaling pathway	Phase II trials showed promising activity in AR-positive TNBC
Epidermal growth factor receptor (EGFR)	Cetuximab, Gefitinib	Inhibits EGFR signaling pathway	Limited efficacy in unselected TNBC patients
PI3K/AKT/mTOR pathway	Everolimus, Alpelisib	Inhibits PI3K/AKT/mTOR signaling pathway	Phase II trials ongoing, with mixed results in TNBC patients

### Immunotherapy in TNBC

Recent advances in immunotherapy have shown promising results for TNBC. The TORCHLIGHT trial revealed that combining toripalimab with nab-paclitaxel significantly extends progression-free survival (PFS) in stage IV breast cancer or recurrent/metastatic TNBC patients (75). Additionally, the FUTURESUPER trial demonstrated improved outcomes with immunotherapy tailored to molecular subtypes, such as Immunomodulatory (IM) TNBC, which constitutes about 24% of TNBC cases (76). Immune checkpoint inhibitors, particularly programmed cell death protein 1 (PD-1) and programmed death-ligand 1 (PD-L1) inhibitors, have emerged as promising therapeutic agents in TNBC (77). Pembrolizumab, an anti-PD-1 inhibitor, demonstrated improved overall survival in TNBC patients with PD-L1-positive Tumors in the KEYNOTE-522 trial, leading to FDA approval in combination with chemotherapy as neoadjuvant therapy (78). Other immune checkpoint inhibitors, such as atezolizumab and durvalumab, are also being evaluated in TNBC clinical trials, either as monotherapy or in combination with chemotherapy (**Table 3**). Combination approaches incorporating immunotherapy with chemotherapy, targeted therapies, or other immunomodulatory agents are being investigated in clinical trials to enhance treatment efficacy and overcome resistance mechanisms in TNBC. Despite these advancements, challenges remain in identifying predictive biomarkers of response and overcoming resistance to immunotherapy in TNBC. Ongoing research efforts aim to elucidate the underlying mechanisms of immunotherapy resistance and develop more effective combination strategies for TNBC patients. Chimeric antigen receptor (CAR) T cell therapy and tumor vaccines are emerging strategies for TNBC. CAR-T therapy targeting ROR1 and MUC1 showed promising antitumor activity in preclinical studies (79). Tumor vaccines, including dendritic cell and peptide vaccines, are under investigation, with ongoing clinical trials assessing their efficacy.

**Table 3:** Immunotherapy in TNBC.

Therapeutic Agent	Mechanism of Action	Clinical Trials and Results
Pembrolizumab (anti-PD-1)	Blocks PD-1/PD-L1 interaction, enhances T-cell-mediated anti-tumor immune response	KEYNOTE-086, KEYNOTE-119 trials demonstrated improved overall response rates and survival in PD-L1-positive TNBC
Atezolizumab (anti-PD-L1)	Blocks PD-L1 interaction with PD-1, enhances T-cell activation and anti-tumor immunity	IMpassion130, IMpassion131 trials showed improved progression-free survival in PD-L1-positive TNBC patients

## Precision medicine approaches

Precision medicine aims to tailor treatment strategies to the unique molecular characteristics of individual Tumors, to improve therapeutic outcomes and minimize adverse effects. In TNBC, precision medicine approaches encompass a variety of strategies, including genomic profiling, targeted therapies, and personalized treatment regimens.

Genomic Profiling: Genomic profiling techniques, such as next-generation sequencing (NGS) and gene expression profiling, provide a comprehensive molecular characterization of TNBC Tumors, enabling the identification of actionable genetic alterations and potential therapeutic targets. By analyzing the mutational landscape of TNBC Tumors, clinicians can stratify patients into molecular subgroups and select appropriate targeted therapies based on Tumor-specific biomarkers (80). For example, Tumors with BRCA1/2 mutations may benefit from treatment with poly (ADP-ribose) polymerase (PARP) inhibitors, while Tumors with PIK3CA mutations may respond to PI3K pathway inhibitors (50). Targeted Therapies: Targeted therapies selectively inhibit specific molecular targets involved in TNBC pathogenesis, offering the potential for enhanced efficacy and reduced toxicity compared to conventional chemotherapy.

## Overcoming treatment resistance

Despite advances in targeted therapy development, treatment resistance remains a significant challenge in TNBC management. Multiple mechanisms contribute to treatment resistance, including Tumor heterogeneity, adaptive signaling pathways, and the Tumor microenvironment (**Table 4**). Strategies to overcome treatment resistance in TNBC include:

**Table 4:** Summary Table of Targeted Therapies and Mechanisms of Action in Triple-Negative Breast Cancer (TNBC).

Therapy	Mechanism of Action
PARP Inhibitors	Inhibit poly (ADP-ribose) polymerase, leading to DNA damage accumulation and synthetic lethality in BRCA mutated tumors
Immune Checkpoint	Block immune checkpoints (e.g., PD-1/PD-L1) to
Inhibitors	enhance T-cell-mediated antitumor immune responses and overcome immune evasion
EGFR Inhibitors	Target epidermal growth factor receptor (EGFR) signaling to inhibit tumor growth and survival pathways
PI3K/AKT/mTOR Inhibitors	Inhibit PI3K/AKT/mTOR signaling pathway, suppressing cell proliferation, survival, and metabolism
CDK4/6 Inhibitors	Block cyclin-dependent kinase 4/6 (CDK4/6) to inhibit cell cycle progression and proliferation in TNBC cells
VEGF Inhibitors	Inhibit vascular endothelial growth factor (VEGF) signaling to suppress angiogenesis and tumor vascularization

Combination Therapies: Combining targeted therapies with conventional chemotherapy or other targeted agents may overcome resistance mechanisms and improve treatment efficacy. Rational combinations targeting multiple signaling pathways or exploiting synthetic lethality have the potential to enhance Tumor cell killing and delay the emergence of resistance (81).

Biomarker-guided Treatment: Identification of predictive biomarkers associated with treatment response can guide therapeutic decision-making and optimize patient outcomes. Integrating genomic profiling, proteomic analysis, and immune profiling may facilitate the identification of predictive biomarkers and the development of personalized treatment regimens tailored to individual patient characteristics (82).

Novel Treatment Modalities: Exploration of novel treatment modalities, such as antibody-drug conjugates (ADCs), bispecific antibodies, and Tumor-targeted nanoparticles, offers new avenues for overcoming treatment resistance in TNBC (83). These innovative approaches aim to deliver cytotoxic agents directly to Tumor cells while minimizing off-target effects, thereby enhancing therapeutic efficacy and reducing treatment-related toxicities.

precision medicine approaches hold promise for improving therapeutic outcomes and overcoming treatment resistance in TNBC. By leveraging genomic profiling, targeted therapies, and innovative treatment modalities, clinicians can tailor treatment regimens to the unique molecular characteristics of individual Tumors, ultimately improving patient outcomes and quality of life. Collaborative efforts among researchers, clinicians, and pharmaceutical companies are essential to advance precision medicine in TNBC and address the unmet clinical needs of patients with this aggressive disease.

## Future directions and challenges

Identification of Novel Targets: Despite advancements, identifying new molecular targets and signaling pathways remains challenging in triple-negative breast cancer (TNBC). The complexity of TNBC biology necessitates thorough exploration using advanced screening techniques and comprehensive genomic profiling. Integration of emerging technologies such as single-cell sequencing and spatial transcriptomics could provide deeper insights into the heterogeneity of TNBC tumors and reveal novel therapeutic targets.

Combination Therapies: While combination therapies hold promise, determining optimal combinations and sequencing remains a challenge (84). Integrating genomic, transcriptomic, and proteomic data to identify robust biomarkers predictive of treatment response and resistance is essential for patient stratification and personalized treatment. Additionally, preclinical models and computational modeling approaches can aid in predicting synergistic effects and guiding clinical trial design for combination therapies.

Biomarker Identification: Defining predictive biomarkers of response to immunotherapy is a major challenge. While PD-L1 expression is associated with response in some Tumors, its utility in TNBC is uncertain (85). Validation of biomarkers such as Tumor mutational burden (TMB) and immune cell infiltrates is necessary. Combination Strategies: Exploring rational combinations of immunotherapy with other modalities is promising but challenging. Determining optimal sequencing, dosing, and scheduling of combination regimens through clinical trials is essential for maximizing treatment efficacy. Overcoming Resistance: Resistance to immunotherapy is a significant hurdle. Understanding underlying resistance mechanisms, including Tumor immune evasion and T-cell exhaustion, is critical for developing strategies to overcome resistance and enhance response rates (86).

Precision Medicine: Integrating multi-omics data provides a comprehensive understanding of TNBC biology, but challenges exist in analyzing complex datasets. Advanced bioinformatics tools and machine learning algorithms are necessary for identifying actionable therapeutic targets and developing predictive models of treatment response. Moreover, collaborative efforts among researchers, data scientists, and bioinformaticians are essential for standardizing data analysis pipelines and sharing insights across research consortia (87).

Clinical Implementation: Translating precision medicine approaches into clinical practice requires overcoming various challenges. Robust validation of biomarkers, standardization of testing protocols, and establishing infrastructure for molecular profiling are essential for the successful implementation of precision medicine in TNBC. Additionally, interdisciplinary collaborations between oncologists, pathologists, genetic counselors, and healthcare administrators are necessary to integrate molecular profiling into routine clinical care and optimize treatment decision-making for TNBC patients.

## Conclusion

In conclusion, triple-negative breast cancer (TNBC) remains a formidable challenge in the field of oncology due to its aggressive behavior, limited treatment options, and poorer prognosis compared to other breast cancer subtypes. Despite significant progress in therapeutic and diagnostic approaches, several challenges persist in TNBC management. Targeted therapies offer hope for personalized treatment strategies in TNBC, but the identification of novel targets, exploration of combination therapies, and development of predictive biomarkers are crucial for improving treatment outcomes. The integration of emerging technologies and comprehensive molecular profiling approaches holds promise for uncovering new therapeutic targets and refining patient stratification strategies. Immunotherapy represents a transformative approach in TNBC treatment, yet challenges in biomarker identification, combination strategies, and overcoming resistance must be addressed to maximize its clinical benefit. Advancements in understanding tumor immune evasion mechanisms and rational combination approaches are essential for enhancing antitumor immune responses and improving patient outcomes. Precision medicine approaches offer opportunities to tailor treatment based on tumor biology, but integration of multi-omics data and clinical implementation present challenges that require innovative solutions. Collaborative efforts among researchers, clinicians, patients, and industry partners are critical for translating scientific discoveries into clinical practice and improving outcomes for TNBC patients. Overall, addressing the complexities of TNBC biology and developing effective therapeutic approaches require sustained interdisciplinary collaborations, continued research efforts, and participation in well-designed clinical trials. By overcoming these challenges and advancing treatment strategies, we can strive towards personalized, effective treatments that improve outcomes and ultimately enhance the lives of TNBC patients. Through collective efforts, we can realize the vision of precision oncology in TNBC management and make meaningful progress towards achieving better patient outcomes and quality of life.

## Author contributions

XN: Conceptualization, Formal analysis, Investigation, Methodology, Writing – original draft, Writing – review & editing. WH: Investigation, Software, Writing – original draft, Writing – review & editing. ZY: Funding acquisition, Software, Supervision, Visualization, Writing – original draft, Writing – review & editing.
